# Antifungal and Immunomodulatory Ingredients from Traditional Chinese Medicine

**DOI:** 10.3390/antibiotics12010048

**Published:** 2022-12-28

**Authors:** Hua Zhong, Lei Han, Ren-Yi Lu, Yan Wang

**Affiliations:** 1 School of Pharmacy, Second Military Medical University, Shanghai 200433, China; 2 School of Pharmacy, Fujian University of Traditional Chinese Medicine, Fuzhou 350122, China

**Keywords:** natural product, traditional Chinese medicine, antifungal drug, immune regulation, combination of drugs, fungal infection

## Abstract

Fungal infections have become a growing public health challenge due to the clinical transmission of pathogenic fungi. The currently available antifungal drugs leave very limited choices for clinical physicians to deal with such situation, not to mention the long-standing problems of emerging drug resistance, side effects and heavy economic burdens imposed to patients. Therefore, new antifungal drugs are urgently needed. Screening drugs from natural products and using synthetic biology strategies are very promising for antifungal drug development. Chinese medicine is a vast library of natural products of biologically active molecules. According to traditional Chinese medicine (TCM) theory, preparations used to treat fungal diseases usually have antifungal and immunomodulatory functions. This suggests that if antifungal drugs are used in combination with immunomodulatory drugs, better results may be achieved. Studies have shown that the active components of TCM have strong antifungal or immunomodulatory effects and have broad application prospects. In this paper, the latest research progress of antifungal and immunomodulatory components of TCM is reviewed and discussed, hoping to provide inspiration for the design of novel antifungal compounds and to open up new horizons for antifungal treatment strategies.

## 1. Introduction

The development of new drugs has a very important role in improving human health and extending human lifespan. There are many strategies for drug development, including screening drugs from natural products and synthetic biology strategies. Traditional Chinese medicine (TCM) is a huge reservoir of natural products, with a long history of practice and great potential for drug development. The discovery of novel drug candidates from TCM and its extracts has become a research hotspot [1].

Fungal infections have been a major public health challenge. Depending on the site of invasion, there are at least three types: superficial, subcutaneous, and deep. Most of these infections are superficial, i.e., non-fatal infections of the skin, nails, and hair, mainly caused by skin fungi, with a global incidence of about 25% [2,3]. Deep mycosis, by contrast, can be life-threatening. The infection can spread to many organ systems and meninges. Under different circumstances, the mortality rate can vary from 35% to 90% [2]. Common pathogens of invasive fungal diseases include *Candida albicans*, *Cryptococcus neoformans*, and *Aspergillus fumigatus* [4,5]. Despite continuous efforts to develop antifungal drugs, the availability of drugs for clinical use remains limited. At present, commonly used antifungal drugs in clinics are azole, polyene, echinomycin, terbinafine, and so on. However, existing drugs have problems such as drug resistance, high price, high toxicity, and serious side effects, such as renal toxicity. It is an urgent and challenging task to find new antifungal drugs with high efficiency and low toxicity.

The TCM treatment of infectious diseases focuses more on the treatment of diseases rather than just killing pathogens. According to the basic theory of TCM, diseases can be divided into *cold*, *heat*, *deficiency*, and *excess*. The meaning of the words mentioned here is different from the literal meaning. For example, “*heat*” refers to a range of different kinds of diseases, which include a range of symptoms caused by an excessive inflammatory response, such as vulvovaginal candidiasis. In the case of “*deficiency*”, diseases caused by immune deficiency are included, such as invasive pulmonary aspergillosis. Thereby, drugs with anti-inflammatory effects are usually used to treat fungal infections with *heat*. These drugs include *Fructus Forsythiae* (lianqiao in Chinese) and *Lonicera japonica* Flos (jinyinhua in Chinese). Meanw hile, some drugs with immune-modulatory effects are used to tonify “*deficiency*”. These drugs include ginseng, *Atractylodes macrocephala* Koidz. (baizhu in Chinese), and *Schisandra chinensis* (Turcz.) Baill. (wuweizi in Chinese). TCM treats the interaction between the fungus and its host as a whole. If we try to explain it in modern medical terms, it could be killing the fungus while modulating the body’s immune response.

There are several TCM products that use both antifungal and immunomodulatory strategies to treat fungal infections. For example, Skinguard Lotion, a TCM product for the treatment of vaginitis, contains *Lonicera japonica* Flos, *Taraxacum officinale* L., *Cnidii Fructus*, etc. The pharmacological effects of the lotion include inhibiting inflammation, relieving itching, and killing pathogens. In the lotion, *Cnidii Fructus* is reported to have antifungal effects [6], while the main active molecules in *Lonicera japonica* Flos and *Taraxacum officinale* L. are proven to have anti-inflammatory effects [7,8]. Patients with vaginitis suffer a lot from persistent itch caused by inflammation response. The lotion can reduce the itching and at the same time inhibit pathogens. It can both treat the symptoms and eliminate the causes of the disease. The combination of antifungal drugs and immunomodulators may be a promising antifungal treatment strategy and an effective way to develop new antifungal drugs. Therefore, this paper reviewed two kinds of substances isolated from TCM: those with antifungal effects and those with immunomodulatory effects.

## 2. Antifungal Ingredients from TCM

Hundreds of TCM have been shown to have antifungal effects, such as *Coptis chinensis* Franch [9], *Scutellaria baicalensis* Georgi [10], *Curcuma longa* Linn [11], and *Allium sativum* L. [12]. We can extract active substances from these TCMs with water, alcohol, chloroform, ethyl acetate, ether, and petroleum ether. The extracts, such as terpenoids, volatile oil, ketones, alkaloids, aldehydes, phenylpropanoids, and saponins, also exhibit potent antifungal activity [13]. We reviewed substances isolated from TCMs with antifungal activities, including dioscin, α-santalol, formyl-phloroglucinol meroterpenoids, asiatic acid, carvacrol, eugenol, thymol, turmeric oil, terpinen-4-ol, silibinin, pinobanksin, tectochrysin, chrysin, licochalcone A, baicalein, baicalin, berberine, sanguinarine, plumbagin, osthole, and ethyl caffeate (Table 1).

### 2.1. Terpenoids

Terpenoids are common substances in natural products. Many terpenoids have important biological activities and are important resources for the development of new drugs. Some terpenoids in natural medicines have important biological activities and have been used in clinical practice for many years, such as the antimalarial drug artemisinin, the anticancer drugs paclitaxel and cantharidin, the antibiotic drug lactam, etc. Some terpenoids have antifungal activity and may provide new strategies for antifungal therapy.

For example, dioscin is a steroidal saponin that can be isolated from TCMs of the *Dioscoreaceae* family. It exhibits significant fungicidal effects against the *Candida* species, such as *C. albicans*, *C. glabrata*, and *C. parapsilosis*, with minimum inhibitory concentration (MIC) at 2–4 µg/mL. Moreover, dioscin can also inhibit biofilm formation and even destroy the mature biofilm at high concentrations [14]. The possible antifungal mechanisms of dioscin may be associated with inhibiting the virulence factors of *C. albicans*, including the morphological transformation, the production of phospholipase, and the adherence to the abiotic surface [14]. Another computational study showed that dioscin can bind to the glucoamylase enzyme that is produced in many fungi by molecular docking [55].

Another example of antifungal terpenoids is α-santalol. It can be found in the essential oil of the *Santalum* species, which has been historically used as a noble perfume as well as medicine. It is reported that α-santalol inhibits Trichophyton rubrum at 12.5 µg/disc (using the disc diffusion method), and a preliminary mechanism study revealed that α-santalol influences the fungal cell wall synthesis and mitosis process [16].

Formyl-phloroglucinol meroterpenoids (FPMs) are important secondary metabolites with various biological activities, mainly found in *Eucalyptus* and *Psidium*. FPMs exhibit antifungal effects against many fungal species, including *C. albicans* and *T. mentagrophytes* [56,57]. *Eucalyptus robusta* is widely distributed in Sichuan, Yunnan, and other southern areas of China, and it is commonly used in the traditional medicine of ethnic minorities. Eucalyptus leaves are used to treat respiratory infections, intestinal infections, malaria, trachoma, otitis media, keratitis, dermatitis, etc. The essential oil extracted from the leaves is also used as a preservative. Eucarobustol E, as an FPM from the leaves of *Eucalyptus robusta*, has a broad-spectrum antifungal effect with MIC ranging from 4 to 16 µg/mL. It also inhibits *C. albicans* biofilms in both the formation phase and mature phase, and the mechanism might involve the negative regulation of hyphal growth due to the inhibition of carbon flow towards ergosterol. This mechanism is totally different from the known mechanisms of existing antifungal drugs and may provide a novel strategy for fungal infection treatment [18].

Carvacrol is a phenolic monoterpenoid that can be extracted from *Origanum vulgare*, an herb that is used to treat acute gastroenteritis in TCM. It inhibits the spore germination of *Aspergillus flavus* by 73.3% at the concentration of 100 µg/mL, and 100 µg/mL of carvacrol slows the mycelia growth and reduces mycelia drying weight. The antifungal mechanism involves deficiency in ergosterol production and the alteration of glycerophospholipid metabolism [58].

Thymol is one of the main components of thyme oil, which is used as both pharmacological and cosmetic additives. The MIC of thymol against *C. albicans* is 500 µg/mL [20]. Thymol also exhibits antifungal activity against *C. neoformans*, with MICs ranging from 20–51 µg/mL, and the mode of antifungal action seems to be related to the ergosterol of yeast [23]. Another study demonstrated that thymol regulated several signaling pathways, such as calcineurin, unfolded protein response, and the HOG-MAPK pathway [59].

In addition to the terpenoids with antifungal effects when used alone, there are also some terpenoids that can be used as synergists in combination with existing antifungal drugs to reduce the dosage and increase efficacy. Doke S. K. et al. evaluated the synergistic effects of three terpenoids with fluconazole against *C. albicans*, involving carvacrol, eugenol, and thymol. The results indicated that carvacrol and eugenol showed synergistic effects in combination with fluconazole against planktonic cells and biofilm formation, with FICI ranging from 0.25 to 0.516, whereas thymol showed an indifferent effect with fluconazole. Using terpenoids in combination with current antifungal drugs, such as fluconazole, may be a feasible strategy to treat fungal infections. The combination can effectively reduce the dosage of antifungal drugs and, therefore, reduce the adverse effects during treatment [20].

Asiatic acid (AA) is a natural ursane-type pentacyclic triterpenoid, mainly found in plants such as *Centella asiatica*. AA shows synergistic effects with fluconazole, and the combination can inhibit fluconazole-resistant *C. albicans* strains in vitro and in vivo, with a fractional inhibitory concentration index (FICI) of 0.25. 32 µg/mL. AA also increases the fluconazole effect against *C. albicans* biofilm at 0.125–0.25 µg/mL. A mechanism study indicated that AA inhibited the drug efflux pump of *C. albicans*. The inhibitory effect of AA on the drug efflux pump may increase the intracellular concentration of fluconazole in *C. albicans* cells, and thereby, AA exhibits synergistic effects with fluconazole. Meanwhile, AA in combination with fluconazole aroused the level of intracellular reactive oxygen species (ROS) and inhibited the hyphal growth of yeast [19].

### 2.2. Volatile Oils

Volatile oils, also known as essential oils, are extracted from plants and animals with various bioactivities. They are an important source for developing antifungal agents. Volatile oils have complex molecular structures and usually have a strong fragrance. According to TCM theories, volatile oils have the functions of inducing diaphoresis, regulating Qi, relieving pain, being antimicrobial, and correcting flavor, whereas modern medical research shows that essential oils have antibacterial, antifungal, antiviral, antioxidant, antitumor, and immunomodulatory activities [60]. It seems that natural medicines with antifungal effects are mostly found in *Lamiaceae*, *Lauraceae*, *Myrtaceae*, and *Compositae*, such as *Cinnamomum cassia* Presl, *Rosmarinus officinalis*, *Ocimum basilicum*, *Origanum vulgare*, *Mentha haplocalyx* Briq., *Melaleuca alternifolia*, *Foeniculum vulgare*, *Zingiber officinale*, *Catsia tora Linn*, *Cinnamomum camphora* (L.) Presl., and *Senecio scandens*.

Abers M. et al. evaluated 19 essential oils for their antifungal activity using a modified disk diffusion test. The results demonstrated that volatiles of lavender, tea tree, cinnamon, peppermint, cassia, and oregano had “moderate” antifungal activity, whereas volatiles of thyme and rosemary had “high” antifungal activity. Clove volatile did not exhibit any antifungal activity in the test [61]. A similar work was conducted by another team. Bona E. et al. tested the sensitivity of 30 vaginal *C. albicans* strains to 12 essential oils, and mint, basil, lavender, tea tree oil, and oregano were more efficient in inhibiting fungal growth and activity than the traditional antifungal drug clotrimazole. The MICs of mint, basil, lavender, and tea tree oil were 0.25–4% *v*/*v* for most strains, with values >4% for others. The oregano volatile MICs were lower than 1% *v*/*v* in 64% of the strains. They also found that the mechanism of essential oils in antifungal activity was associated with cell wall and membrane damage [25].

Turmeric oil is the extract from *Curcuma longa* Linn. It has long been used as a common household medicine and a yellow spice in Southeast Asia. As for antifungal activities, turmeric oil completely inhibits common dermatophyte growth at the concentration of 0.2% *v*/*v*, including *Epidermophyton floccosum*, *Microsporum gypseum*, *M. nanum*, *T. mentagrophyte*, *T. rubrum*, and *T. violaceum* [29]. Notably, turmeric oil exhibits low toxicity towards humans. It did not show any irritation or adverse effect at a 5% concentration for up to 3 weeks in a clinical trial [29]. Moreover, the main component of turmeric oil, Ar-turmerone, exhibits more potent antifungal activity against dermatophytes than ketoconazole, with MICs ranging from 3.90 to 7.81 μg/mL [30]. Another important substance isolated from *Curcuma longa* L. is curcumin, which also arouses the wide interest of scientists. It is reported to have many bioactivities, such as antitumor, antioxidant, anti-inflammatory, and antimicrobial activities [62,63,64]. Curcumin shows inhibitory effects against many common fungal pathogens. It inhibits *Candida* species growth with an MIC_90_ ranging from 250 to 650 μg/mL. This includes clinical isolates and fluconazole-resistant strains of *C. albicans*, *C. glabrata*, *C. krusei*, *C. tropicalis*, and *C. guilliermondii* [32]. Curcumin also shows antifungal activity against *C. neoformans* and *C. dubliniensis* with an MIC of 32 μg/mL [34]. In addition, curcumin and its derivatives show synergy effects with fluconazole against resistant *C. albicans*, *C. tropicalis*, and *C. krusei*, with the FICIs ranging from 0.078 to 0.563 [33]. The antifungal mechanisms of curcumin include causing oxidative stress and inhibiting thymidine uptake 1. Curcumin also alters the membrane-associated properties of ATPase activity, ergosterol biosynthesis, and proteinase secretion [33].

Similar to terpenoids, volatile oils can also be used as a synergist with antifungal drugs. Adding tea tree oil at a concentration of 1/4 MIC can effectively reduce MICs of fluconazole against resistant *C. albicans* strains. Among all the 32 tested strains, the average fluconazole MIC is reduced from 244.0 µg/mL to 38.46 µg/mL, and terpinen-4-ol, which is the major bioactive component of tea tree oil, shows an even stronger inhibition effect [27].

Moreover, a combination of different TCMs without antifungal drugs can also have a synergistic effect against fungi. Apart from enhancing antifungal activity and reducing adverse effects, this kind of combination treatment can also reduce the occurrence of drug resistance in fungi. It is reported that an essential oil blend of *Cinnamomum zeylanicum*, *Daucus carota*, *Syzygium aromaticum*, and *Origanum vulgare* shows antifungal activity against *C. albicans*, *C. tropicalis*, and *C. glabrata*, including strains resistant to fluconazole or amphotericin B, with MICs ranging from 0.01 to 0.05% *v*/*v* [35].

### 2.3. Flavonoids

Some flavonoids from TCMs also have antifungal properties. *Rosmarinus officinalis* L., for example, contains phenols and flavonoids in its extract. The extract shows an antibiofilm effect against *C. albicans*, *C. dubliniensis*, *C. glabrata*, *C. krusei*, and *C. tropicalis*. A 50–200 mg/mL extract can reduce mature biofilms, which are formed 48 h in advance, and the antibiofilm activity of *Rosmarinus officinalis* L. extract is comparable to that of nystatin [26].

Silibinin is a flavonoid that can be found in *Silybum marianum*, a traditional medicine for hepatobiliary diseases in China and Europe [65]. The antifungal activity of silibinin seems not stable or uniform. Dayanne R. O. et al. reported that silibinin inhibits *C. albicans*, *C. krusei,* and *C. tropicalis* with MICs at 1024 µg/mL [36]. In another study, however, the MICs of silibinin against *C. albicans* and *C. parapsilosis* were 19.3 µg/mL and 9.6 µg/mL, respectively [37]. One possible reason for the differences is that the strains used in the tests are different and silibinin does not exhibit stable or uniform antifungal activities against those strains. Silibinin at 100 µg/mL also inhibits *C. albicans* biofilm formation to approximately 50%, and the mode of action involves causing membrane damage to fungal cells [65].

Propolis is a natural product with antimicrobial [66], anti-inflammatory [67], and antioxidant activities [68]. The main bioactive components of propolis are flavonoids. Propolis production is influenced by many environmental factors such as local climate and the plants nearby. Therefore, propolis from different regions contains different kinds and amounts of flavonoids, and it consequently has different bioactivities [69]. In Chinese propolis, the four major flavonoids are pinobanksin, tectochrysin, chrysin, and 3-O-acetylpinobanksin. All the four flavonoids have antifungal activities against *C. albicans*, with MICs of 100 µg/mL, 25 µg/mL, 100 µg/mL, and 50 µg/mL, respectively [39]. The antifungal mechanism of propolis is related to the induction of apoptosis through metacaspase and Ras signaling. It is also reported that propolis disrupts the expression of several genes involving pathogenesis, cell adhesion, biofilm formation, filamentous growth, and phenotypic switching [70].

The *Glycyrrhiza* species is another commonly used ingredient in traditional herbal remedies. Licochalcone A is a bioactive natural product isolated from *Glycyrrhiza* species, and it exhibits antifungal activities against *C. albicans* with MICs ranging from 16.92 µg/mL to 50.76 µg/mL. Licochalcone A also significantly inhibits *C. albicans* biofilm growth at 10 × MIC, and an in vivo experiment conducted in mice with oral candidiasis indicated that licochalcone A decreases numbers of fungal colonies in tongue tissue. The mechanism study revealed that licochalcone A decreased proteinases and phospholipases secreted by *C. albicans* biofilm [40].

The combination of flavonoids and antifungal agents can exhibit synergy. The major active components of TCM *Scutellaria baicalensis* are baicalein and baicalin [71]. Both of the two major components exhibit antifungal activities. The MIC of baicalein is 25 µg/mL [41]. It induces the apoptosis of *C. albicans* cells and has potent synergy with fluconazole against resistant strains, with an FICI of 0.039 [42]. The derivatives of baicalein even show greater synergistic antifungal effects with FICIs < 0.007 [72]. There is also evidence that baicalein reduces the MIC of amphotericin B with an FICI ranging from 0.031 to 0.677, and further mechanism investigation indicated that the combination of the two agents accelerates *C. albicans* apoptosis [73].

### 2.4. Alkaloids

Alkaloids are a group of cyclic nitrogen-containing small molecules with diverse bioactivities. They are abundant natural products and provide many valuable molecules in the medical field, such as morphine, quinine, vinblastine, and berberine.

Berberine has been used for treating diarrhea for thousands of years in China and is still used widely in contemporary medicine. There are many studies on the antifungal effects of berberine. It is reported that berberine inhibits the *Candida* species, i.e., *C. albicans*, *C. krusei*, *C. glabrata*, and *C. dubliniensis*, with MICs ranging from 10 to 160 µg/mL [43]. The antifungal activity of berberine alone is unstable and weak in some situations. Therefore, researchers have turned their attention to combining berberine with other drugs. Our group members found that berberine showed potent synergy with fluconazole against fluconazole-resistant *C. albicans* strains. We tested 40 clinical isolates, and the median FICI was 0.034 (range, 0.017 to 0.127) [44]. Further mechanism investigation showed that berberine played a major role in killing the fungi in the synergy, and fluconazole increased the intracellular berberine concentration by inhibiting ergosterol synthesis in cell membranes [74,75]. Berberine also exhibits synergy with other antifungal drugs such as amphotericin B and terbinafine. It was reported that the combination of berberine and amphotericin B reduced approximately 75% of the amphotericin B dose in a mouse model [76], and 100 µg/mL of berberine can effectively assist the antifungal potential of terbinafine [77]. Berberine inhibits biofilms of the *Candida* species in a dose-dependent manner, and 40, 5120, 320, 40, and 1280 μg/mL of berberine can inhibit biofilms of *C. albicans* ATCC 10231, *C. albicans* ATCC 90028, *C. krusei* ATCC 6258, *C. glabrata* ATCC 90030, and *C. dubliniensis* MYA 646, with inhibition rates of 43.54 ± 1%, 19.89 ± 0.57%, 96.93 ± 1.37%, 92.36 ± 0.32%, and 21.62 ± 0.51%, respectively [43].

Sanguinarine and chelerythrine are two alkaloids with similar structures. Both of them can be isolated from *Papaveraceae* plants and have diverse bioactivities. They share similar antifungal activities against *C. albicans* and *C. neoformans* with MICs of 4 µg/mL and 64 µg/mL, respectively, and a 1:1 mixture of sanguinarine and chelerythrine exhibits stronger antifungal effects with an MIC of 2 µg/mL for *C. albicans* and 16 µg/mL for *C. neoformans* [45]. Another study reported that sanguinarine inhibited 10 *C. albicans* strains, with the MICs within the range of 37.5–50 µg/mL. Sanguinarine also showed anticandidal effects in a murine model at the dose of 1.5 and 2.5 mg/kg, and the mode of action is related to ergosterol synthesis deficiency in cells [46]. Sanguinarine can also inhibit *C. albicans* biofilms, and 3.2 µg/mL of sanguinarine significantly inhibits *C. albicans* biofilm formation by over 90% and destroys mature biofilms by 68.3%. The possible mechanism of sanguinarine’s antibiofilm effect involves its inhibitory effect on adhesion and hypha formation due to cAMP pathway suppression [78]. In addition to *C. albicans* and *C. neoformans*, the antifungal spectrum of chelerythrine consists of *C. glabrata*, *C. krusei*, and *C. tropicalis*. According to Gong et al., the MICs of chelerythrine against *C. albicans*, *C. glabrata*, *C. krusei*, and *C. tropicalis* are 2, 16, 16, and 8 µg/mL, respectively, and the mechanism investigation showed that chelerythrine inhibited hyphal growth, increased intracellular calcium concentration, induced the accumulation of intracellular ROS, and inhibited drug transporter activity [47].

*Piper betle* L. var. nigra is a plant used as a spice and medicine belonging to the *Piperaceae* family. It contains many bioactive chemicals such as cadinene, caryophyllene, and amide alkaloids [79,80], and the extract of *P. betle* showed inhibitory effects against *C. albicans* according to a disk diffusion test [81].

### 2.5. Quinones

Quinones are six-membered α,β-dienonic rings that occur in nature. Naphthoquinones is one of the most common classes in quinones. Futuro D.O. et al. reviewed the development of naphthoquinones as antifungal agents against the *Candida* species and identified 30 naphthoquinones with better antifungal activities than those of the existing drugs [82]. Plumbagin, for example, exhibits antifungal effects against *C. albicans* ATCC 10,231, with an MIC of 0.78 µg/mL [48,82]. In a recent study, plumbagin exhibited antifungal effects against *C. neoformans* with an MIC of 8 µg/mL. Further study showed that plumbagin disrupted cell membrane integrity and reduced metabolic activities of the pathogen. It also damaged formation-phase and mature-phase biofilms of *C. neoformans* at concentrations of 64 and 128 µg/mL, respectively. A mechanism study confirmed that plumbagin damaged biofilm by down-regulating FAS1 and FAS2 expression [49], and bis-naphthoquinone, which can be extracted from *Ceratostigma plumbaginoides*, has an MIC of 0.09 μg/mL against *C. albicans* ATCC 25,555 [50].

Another example is shikonin; it has fungicidal activity against *C. albicans* with MICs of 2–8 μg/mL [51,52], and it exhibits antifungal activity against other *Candida* species, *C. neoformans*, *T. cutaneum*, and *Saccharomyces cerevisiae*, with MICs ranging from 4 to 16 μg/mL (see details in Table 1) [51]. Using metabonomics, shikonin was found to boost histone H3 on lysine 56 residue acetylation via *HST3* in *C. albicans* and execute its antifungal activity [83]. Shikonin can also inhibit the formation of biofilms and destroy the maintenance of mature biofilms at concentrations of 1–32 µg/mL and 4–16 µg/mL, respectively. This antibiofilm activity was confirmed in a mouse vulvovaginal candidiasis model. The possible mechanisms involve down-regulating hypha- and adhesion-specific gene expression and inducing farnesol production [52].

### 2.6. Coumarin

Osthole is a natural coumarin that can be isolated from TCM *Cnidii fructus*. It does not show antifungal activity against *C. albicans* at up to 64 μg/mL. However, 1–16 µg/mL of osthole has significant synergy with fluconazole against fluconazole-resistant *C. albicans*, with FICIs ranging from 0.04 to 0.37 [6,53], and further study indicated that the mechanism of synergy is associated with endogenous ROS accumulation [53].

Designing novel coumarin derivatives is another common way of developing antifungal agents, since coumarin has many modifiable sites. Linking other pharmacophores, such as azole and quinoline, to coumarin molecules can effectively expand the antimicrobial spectrum and enhance antifungal activity [84].

### 2.7. Others

Ethyl caffeate (EC) can be extracted from *Elephantopus scaber* L., which is widely distributed in the southwest of China and used as TCM, treating fevers and microbial infections [85]. EC exhibits antifungal effects against 26 isolates of *C. albicans*, with MICs ranging from 64 to 256 µg/mL, and the combination of EC and fluconazole shows synergism in 14 out of 26 tested isolates, with FICIs ranging from 0.047 to 0.375. Similar results are observed in *C. albicans* biofilms. EC shows no antibiofilm effects at up to 256 µg/mL. However, EC and fluconazole exhibit synergy against 11 out of 26 isolates, with FICIs ranging from 0.002 to 0.375. The synergy mechanisms may be related to the inhibition of hydrolase secretion and drug efflux function of *C. albicans* [54].

## 3. Immunomodulatory Ingredients from TCM

The immune modulatory effect of TCM on fungal infection include the inhibition of inflammation and the enhancement of fungal clearance. According to TCM theory, most herbs with anti-inflammatory effects fall under the category of “*heat-clearing*” drugs, including *Fructus Forsythiae*, *Lonicera japonica* Flos, and *Menthae Haplocalycis Herba*. This Chinese medicine is used to treat inflammatory diseases, and active ingredients of these herbs include phenolic acids, flavonoids, volatile oils, lignans, etc. Meanwhile, *Panax ginseng* C. A. Mey., and *Astragalus membranaceus* are herbs that can improve the fungal clearance function of the immune system. The main active ingredients are polysaccharide, glycoside, alkaloid, volatile oil, and so on. In this section, the common TCM components with immunomodulatory effects were classified according to their chemical structures.

### 3.1. Phenolic Acids

Phenolic acids are widely found in *Lonicerae* and are the main active ingredients in TCM *Lonicera japonica* Flos and *Lonicera japonica Caulis* (both originated from *Lonicera japonica* Thunb.). *Lonicera japonica* Flos is the most important ingredient in many TCM preparations used to treat inflammatory diseases. Its extract shows protective activity against LPS-induced lung inflammatory cytokine release. The mechanism study revealed that the *Lonicera japonica* Flos extract increases nuclear Sp1 binding activity through the incremental phosphorylation of ERK, and it consequently enhances the expression of IL-10. At the same time, the extract suppresses the phosphorylation of IκB, p38, and JNK, thereby inhibits nuclear NF-κB binding activities and down-regulates the expression of TNF-α, IL-1β, and IL-6 in the lung [86].

**Figure 1:**
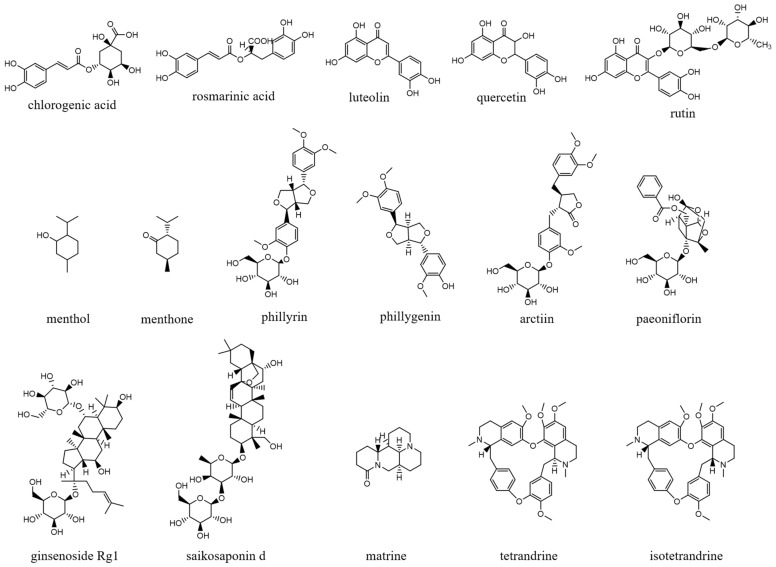
Chemical structures of the natural products isolated from TCMs with immunomodulatory effects.

Chlorogenic acid is one of the main active substances of *Lonicera japonica* Flos (the chemical structures can be found Figure 1). It is reported that chlorogenic acid exhibits anti-proliferation activity against the fibroblast-like synoviocyte cell line (RSC-364), which is stimulated by IL-6. The main mechanism involves JAK/STAT and NF-κB signaling cascades. Chlorogenic acid inhibits these two pathways by suppressing the expression of p-STAT3, JAK1, p50, and IKK, therefore inducing apoptosis in RSC-364 [7]. Interestingly, a recent study revealed that chlorogenic acid also induces apoptosis in fluconazole-resistant *Candida* spp. and exhibits antifungal activity. Molecular docking demonstrates that chlorogenic acid binds to several important drug targets, including thymidylate kinase, CYP51, and ALS3 [87].

Rosmarinic acid (Figure 1) can be found in *Menthae Haplocalycis Herba* and *Prunella vulgaris* [88,89]. Rosmarinic acid has been reported to have anti-inflammatory effects in many diseases, such as arthritis, colitis, atopic dermatitis, asthma, allergic rhinitis, and periodontal disease [90]. It also inhibits LPS-induced inflammation in RAW264.7 cells. The anti-inflammation target of rosmarinic acid involves the NF-κB/MAPK pathway. It suppresses the activation of the NF-κB/MAPK pathway and thereby reduces the production of pro-inflammatory cytokines NO, TNF-α, IL-1β, and IL-6 [91].

### 3.2. Flavonoids

Flavonoids can be found in many TCMs with anti-inflammatory effects, such as *Taraxacum officinale* L., *Fructus Forsythiae*, and *Menthae Haplocalycis Herba*. Common flavonoids in these herbs are luteolin, quercetin, rutin, etc. The extract of *Taraxacum officinale* L., which contains luteolin (Figure 1), was confirmed to have anti-inflammatory effects in many studies. It can reduce the release of inflammatory cytokines, including NO, PGE2, IL-1β, IL-6, and TNF-α, by suppressing the NF-κB/iNOS and MAPK pathway [8,92,93,94]. Research on the anti-inflammatory effects of luteolin dates back at least 20 years [95]. Luteolin can reduce LPS-induced inflammation both in vivo and in vitro. A mechanism study showed that luteolin inhibits the pro-inflammatory molecules TNF-α and ICAM-1 expression in mice [95], and in RAW264.7 cells, luteolin also inhibits NO, IL-1β, and IL-6 in addition to TNF-α [96].

Quercetin (Figure 1) is another common flavonoid in these “*cold*-natured” herbs. It also can be found in some vegetables and fruits. The anti-inflammatory effect of quercetin has been reported in many diseases such as inflammatory bowel disease, multiple sclerosis, asthma, and atherosclerosis, in vivo or in vitro [97,98]. It was also in a clinical trial for treating rheumatoid arthritis [99]. Quercetin inhibits inflammation via promoting anti-inflammatory cytokine secretion (for example, IL-10), reducing pro-inflammatory cytokine release (TNF-α, IL-1β, and IL-6), inhibiting cyclooxygenase and lipoxygenase expression, and maintaining mast cell stability [97,100].

Rutin (Figure 1) is a flavonoid that exists in *Fructus Forsythiae* [98]. Rutin shows anti-inflammatory effects in activated human neutrophils, through inhibiting TNF-α and NO production, as well as myeloperoxidase (MPO) activity [101]. Rutin also attenuates advanced glycation end product induced inflammation on human chondrocytes. The study revealed that rutin targets BCL-2 and TRAF-6 in the NF-κB/MAPK pathway to inhibit inflammation and treat osteoarthritis [102].

### 3.3. Volatile Oils

Volatile oils are the main active ingredients in *Menthae Haplocalycis Herba*, consisting of menthol, menthone, isomenthone, piperitone, linalool, carvone, limonene, α-pinene, β-pinene, etc. The volatile oils of *Menthae Haplocalycis Herba* are reported to exhibit anti-inflammatory and antimicrobial effects, as well as alleviate mental fatigue [88]. Menthol (Figure 1) can be beneficial in rats with acetic acid-induced acute colitis. The protective effect is related to the inhibition of MPO, as well as the reduction of TNF-α, IL-1β, and IL-6 [103]. Menthone (Figure 1) is reported to reduce LPS-induced inflammation in mice. The possible mechanism is that menthone inhibits the activation of NLRP3 inflammasome and consequently reduces the release of pro-inflammatory cytokines, including IL-18, IL-1β, IL-5, TNF-α, IFN-γ, G-CSF, GM-CSF, and MIP-1β [104].

### 3.4. Lignans

Lignans are one of the main active ingredients in *Fructus Forsythiae*. *Fructus Forsythiae* is the dried fruit of *Forsythia suspensa* (Thunb.) Vahl, family *Lignanaceae*. *Fructus Forsythiae* is often used for acute colds belonging to “*heat*”, lymphatic tuberculosis, urinary tract infections, etc.

One of its main lignans with an anti-inflammatory effect is phillyrin (Figure 1). Phillyrin inhibits inflammation both in vivo and in vitro. In a zebrafish model, phillyrin reduced inflammation in a dose-dependent manner and improved survival. A mechanism study showed that phillyrin inhibits the MyD88/IκBα/NF-κB signaling pathway, but not ERK1/2 MAPKs or JNK MAPKs. It reduces neutrophil infiltration and down-regulates the release of IκBα, TNF-α, IL-1β, and IL-6 [105]. By regulating NF-κB signaling, phillyrin also alleviates inflammation induced by SARS-CoV-2 in Huh-7 cells. It decreases the release of pro-inflammatory cytokines including TNF-α, IL-6, IL-1β, MCP-1, and IP-10 [106].

Phillygenin (Figure 1) is another lignan in *Fructus Forsythiae* that targets the NF-κB signaling pathway and shows anti-inflammatory effects. It inhibits LPS-induced inflammation in LX2 and RAW 264.7 cell lines. Molecular docking indicated that phillygenin has an affinity for many proteins in the NF-κB pathway, such as IKKβ, p65, IκBα, and TAK1 [107,108].

Arctiin (Figure 1) can be found in *Fructus Forsythiae* and exhibits anti-inflammatory effects as well. It attenuates inflammation in different cells by inhibiting COX-2 expression, which is an essential protein in inflammation [109,110]. Arctiin also activates Nrf2/HO-1 signaling and blocks the RIG-I/JNK MAPK signaling of A549 cells in inflammation induced by H9N2 avian influenza virus [110]. In an LPS-induced acute lung injury mice model, arctiin significantly ameliorated lung histopathological changes and decreased lung MPO activity. The mechanism study suggested that arctiin targets the PI3K/AKT/NF-κB signaling pathway by inhibiting PI3K/Akt phosphorylation and NF-κB activation [111].

### 3.5. Alkaloids

Some alkaloids have a regulatory effect on the immune function of the body by targeting inflammation-related pathways such as the NF-κB signaling pathway. Matrine, for example, is a tetracyclo-quinolizindine alkaloid (Figure 1) extracted from *Sophora flavescens*. It balanced the Th1/Th2 axis and improved rheumatoid arthritis in a rat model. By regulating the NF-κB pathway, matrine reduced the level of Th1 cytokines (IFN-γ, TNF-α, and IL-1β) and raised Th2 cytokines (IL-4 and IL-10) [112].

Tetrandrine is an isoquinoline alkaloid (Figure 1) that can be isolated from *Radix Stephaniae Tetrandrae*. It is reported to have inhibitory effects on the proliferation of T cells via the NF-κB pathway. Tetrandrine prevents the degradation of IκBα and inhibits nuclear translocation of p65 by blocking IKKα and IKKβ activities, and tetrandrine down-regulates the activation of MAPK including JNK, p38, and ERK, as well as the downstream transcription factor AP-1 [113]. Isotetrandrine has a chemical structure very similar to tetrandrine, differing only in the stereochemistry at the chiral centers (Figure 1). Isotetrandrine has stronger inhibitory effects against the proliferation of T cells than those of tetrandrine [114].

### 3.6. Polysaccharides

Polysaccharides are sugar chains consisting of at least 10 monosaccharides bound by glycosidic bonds. They are complex mixtures and one of the main active ingredients of tonic herbs in TCM, such as polysaccharides from *Ganoderma*, *Astragalus*, *Ginseng*, *Angelica sinensis*, etc. The pharmacological effects of polysaccharides on immune systems are complicated. Some polysaccharides have an inhibiting effect on the immune system to attenuate excessive immune responses, while others have a stimulating effect on the immune system to help the body fight against infections or tumors. In many cases, some polysaccharides have both of these effects. It seems that these polysaccharides can balance immune cells and restore immune functions from abnormal.

*Ganoderma lucidum* polysaccharide is extracted from sporoderm-removed spores of the fungus. On one hand, it inhibits inflammation in AOM/DSS-induced colitis. *Ganoderma* polysaccharide suppresses TLR4/MyD88/NF-κB signaling, inhibits macrophage infiltration, and down-regulates IL-1β, iNOS, as well as COX-2 expressions in the colon. It also inhibits LPS-induced inflammation markers and MAPK activation in RAW264.7, HT-29, and NCM460 cells [115]. On the other hand, *Ganoderma* polysaccharide activates the immune responses by binding to dectin-1, TLRs, MR, or CR3 on immune cells including monocytes, macrophages, dendritic cells (DCs), granulocytes, neutrophils, and natural killing (NK) cells. It also can directly activate lymphocytes and neutrophils [116].

### 3.7. Glycosides

Glycosides can be found in many TCMs such as *Paeonia lactiflora * Pallas, *Radix Ginseng*, *Radix Scutellariae*, *Radix Bupleuri*, etc. As with polysaccharides, the immunomodulatory effects of glycosides are complicated, encompassing both promotive and inhibitory effects.

Paeoniflorin (Figure 1) is a monoterpene glucoside that is the major active component of *Paeonia lactiflora* Pallas. Paeoniflorin has protective effects on many autoimmune diseases in animal models, including arthritis, liver injuries, allergic contact dermatitis, Sjögren syndrome, psoriasis, multiple sclerosis, and asthma [117]. Paeoniflorin regulates the activation of T lymphocytes, B lymphocytes, and macrophages. It also inhibits DC maturation and pro-inflammatory mediator production. As for pathways, paeoniflorin inhibits the MAPK signaling pathway, the JAK2/STAT3 pathway, and the PI3K/Akt/mTOR pathway in immune cells [117].

Ginsenoside Rg1 (Figure 1) is one of the glycosides in *Radix Ginseng*. It has various immune-modulating activities, for example, enhancing the immune activity of Th cells. Ginsenoside Rg1 can help mice fight against disseminated candidiasis. Ginsenoside Rg1 has no antifungal activity against *C. albicans* in vitro. However, it can promote CD4^+^T cell immune response mediated by Th1 cells in infected mice and consequently induces cytokine release including IFN-γ, IL-2, IL-4, and IL-10, exhibiting protective effects in mice [118].

Saikosaponin d (Figure 1) can be found in *Radix Bupleuri* and is one of the major bioactive components of medicine. Saikosaponin d helps generate functional mature neutrophils in cancer chemotherapy-induced neutropenia. The generated neutrophils are capable of resisting infection both in vitro and in vivo. This immune enhancing effect is mediated by the CBL-ERK1/2 pathway, resulting in neutrophil differentiation [119].

## 4. Discussion

Fungal infections can disrupt homeostasis in a number of ways. On the one hand, the invasive growth of fungi destroys mucous membranes and destroys the structure of tissues and organs. On the other hand, it can induce inflammation and trigger dysfunction in the body, making it harder to recover.

In the theory of TCM, the philosophy of treating fungal diseases does not just focus on antifungal, but on both antifungal and immune regulation. Herbal preparations are commonly used to treat fungal diseases, and these often contain a variety of ingredients that have complex regulatory effects on both the fungus and the host. Some ingredients kill the fungus, and others balance the host’s immune response. TCM regulates the host immune response in two directions. Some herbs suppress the immune response by reducing inflammation, while others boost the immune system’s ability to clear fungi. This overall regulation of the fungus and the host is very helpful in the treatment of fungal infections.

However, TCM has two drawbacks in treating fungal infections. First, the effective ingredients of TCM are not clear. Second, the mechanism of TCM treatment of fungal infection is still unclear. In recent years, efforts have been made to explore the active ingredients of TCM and elucidate its mechanism of action. The material basis and mechanism of TCM treatment of fungal infection were discussed in this paper.

### 4.1. Material Basis of TCM for Treating Fungal Infection Diseases

Overall, there are two strategies for TCM to treat fungal infections, antifungal and immunomodulation. There are many chemicals from TCM exhibiting antifungal activities, and most of them can be divided into terpenoids, volatile oils, flavonoids, alkaloids, quinones, coumarin, etc. The terpenoid family of antifungal compounds varies greatly in structure, from thymol, with a molecular weight of less than two hundred, to dioscin, with a molecular weight of more than eight hundred. Interestingly, it seems that the larger the molecular weight of the terpenoid, the better its antifungal activity. Dioscin has the most complex molecular structure in this group, and it has the best antifungal activity against *Candida* spp., with MIC values of 2–4 μg/mL. Volatile oils have their own advantages and disadvantages. Most volatile oils have a pleasant aromatic scent and can be used for environmental sterilization or topical medications. However, their volatile nature makes them difficult to store and transport and are therefore not an ideal antifungal drug preparation. The flavonoids mentioned in this review share a moderate activity against *C. albicans*.

The chemicals with the strongest antifungal activity are from the alkaloid and quinone families, such as chelerythrine and shikonin. They both exhibit broad and potent activities against common *Candida* spp., with MICs ranging from 2 to 16 μg/mL. One potential problem with alkaloids and quinones is their toxicity [120]. Quaternary ammonium alkaloids, such as chelerythrine, bearing a quaternary nitrogen atom, have oxidative effects and are thus usually toxic to cells. An evaluation of the acute hepatotoxicity effect of chelerythrine at a dose of 10 mg/kg/day (i.p.) showed that chelerythrine caused marked parenchymal damage in the liver [121]. Meanwhile, EC_50_ of shikonin against V79 cell lines was 0.4 μg/mL by an MTT assay [122]. Through structural modification, chemicals of these two families are promising antifungal lead compounds.

The single use of Chinese medicine has a significant antifungal effect, and the combination of Chinese medicine and existing antifungal drugs can produce synergistic effects, such as the combination of berberine and fluconazole. Using this synergy, we can enhance the effect of the drug and reduce drug dosage. Therefore, we can reduce the side effects of drugs. In addition, the combination of two or more drugs can also reduce the development of resistance.

Unlike TCM ingredients with antifungal effects, ingredients with immunomodulatory effects have their own unique chemical structure characteristics. Major TCM ingredients with immunomodulatory activity can generally be classified into the categories of phenolic acids, flavonoids, volatile oils, lignans, polysaccharides, glycosides, and alkaloids. There are two types of immunomodulatory effects of these TCM ingredients. Most of the phenolic acids, flavonoids, volatile oils, lignans, and alkaloids show anti-inflammatory effects in the immune response of the host, while polysaccharides and glycosides usually have immune-promoting properties. For example, *Ganoderma lucidum* polysaccharide activates immune response by binding to receptors on immune cells, ginsenoside Rg1 enhance the immune activity of Th cells, and saikosaponin d helps generate functional mature neutrophils in neutropenia hosts.

*Panax ginseng* is a traditional Chinese valuable herb with a history of application for more than 2000 years. Ginseng is used in Chinese medicine to prolong the life of critically ill patients and is used in folklore as a tonic for strengthening the body. Modern pharmacological studies have shown that it has immune-enhancing and antioxidant properties [123]. Polysaccharides and glycosides are two major active components in ginseng, both of which are very promising immunomodulatory drug candidates [124]. They are relatively safe in vivo [125]. Polysaccharides are a relatively complex mixture of components and are therefore difficult to synthesize in vitro, and biosynthesis may be a good way to develop such drugs.

### 4.2. Mechanism of Chinese Medicine in Treating Fungal Infectious Diseases

**Figure 2:**
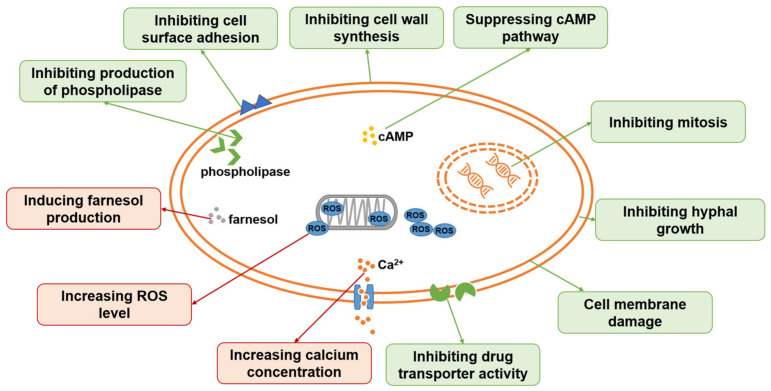
Antifungal mechanisms of some TCM ingredients.

The mechanism of antifungal action of TCM ingredients has been insufficiently studied (Figure 2). Most of the currently reported chemicals do not have a clear target. For the TCM ingredients that act on fungi, the main mechanisms involve inhibiting the virulence factors of fungi (hyphal growth, cell adhesion, production of phospholipase), disturbing fungal cell wall synthesis, damaging the fungal cell membrane, inhibiting the mitosis process, altering the glycerophospholipid metabolism, influencing the drug efflux function, increasing intracellular ROS levels, and inducing apoptosis. Two related pathways in fungal cells are the HOG-MAPK pathway and the Ras-cAMP pathway. Among those chemicals, shikonin is reported to promote histone H3 on lysine 56 residue acetylation via *HST3* in *C. albicans* [83]. Again, shikonin exhibits strong antifungal activities with a broad spectrum and a clear binding target, making it a good candidate to become a lead compound in antifungal drug development.

**Figure 3:**
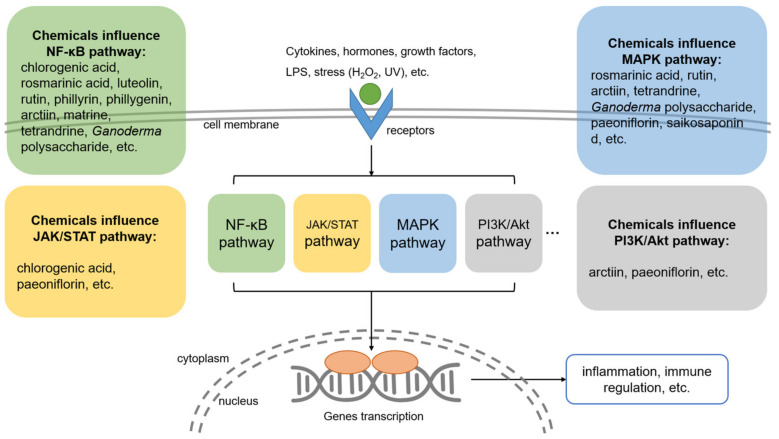
Immunomodulatory mechanisms of some TCM ingredients. When cytokines, hormones, growth factors, or LPS bind to the receptors on the cell membrane, down-stream pathways are stimulated. Transcription factors that are regulated by these pathways enter the nucleus and promote the expression of inflammatory factors or immunomodulatory factors. TCM ingredients affect these pathways and thereby regulate inflammatory processes as well as the immune system.

As for immunomodulatory TCM ingredients, the mechanisms involve two aspects: anti-inflammation and immune enhancement. The pathways that anti-inflammatory ingredients related with are the NF-κB pathway, MAPK signaling pathways, and the JAK/STAT pathway. The NF-κB pathway is one of the most important pathways that regulates inflammation responses in hosts. NF-κB remains inactive in the cytoplasm when binding to its inhibitor IκB. When the cell is stimulated, IKK promotes the phosphorylation of IκB and leads to the dissociation of NF-κB. Then, NF-κB is translocated into the nucleus and binds to DNA, resulting in gene transcription and protein synthesis, which are related to inflammation response [126,127]. Nearly all the anti-inflammatory functions of TCMs listed here are related to the NF-κB pathway (Figure 3). MAPK cascades mediate the signal transduction from extracellular signals to intracellular reactions [126]. There are at least three MAPK cascade signal transduction pathways that are involved in the anti-inflammation effects of TCM ingredients: ERK, JNK, and p38. Rosmarinic acid, rutin, and arctiin down-regulate protein expressions in the MAPK pathway. This reduces inflammatory responses. The JAK/STAT pathway is another type of cascade that translates extracellular chemical signals into the permission of JAK phosphorylation and STAT activation. The pathway is related to many cellular processes, including cell proliferation, differentiation, apoptosis, and immune regulation [128]. Chlorogenic acid and paeoniflorin can inhibit the JAK/STAT pathway and alleviate the inflammation response. However, both the MAPK pathway and the JAK/STAT pathway are involved in many essential biological processes, and this prevents the two pathways from being good anti-inflammatory targets. Inhibiting targets in these pathways may cause many adverse effects.

The immune-enhancing effects are usually induced by stimulating immune cells. For example, *Ganoderma* polysaccharide boosts immune response by binding to dectin-1, TLRs, MR, or CR3 on immune cells. Ginsenoside Rg1 promotes the CD4^+^T cell immune response mediated by Th1 cells, and saikosaponin d helps produce mature neutrophils mediated by the CBL-ERK1/2 pathway.

In addition, there are some interesting molecules, such as chlorogenic acid, that possess both antifungal and anti-inflammatory effects via different mechanisms. On one hand, it possibly binds to CYP51 as well as ALS3 and induces apoptosis in *Candida* spp. On the other hand, it inhibits the JAK/STAT and NF-κB pathways by suppressing p-STAT3, JAK1, p50, and IKK in host cells. This kind of molecule with multiple targets is also a good candidate for drug discovery.

### 4.3. Exploitation of TCM and Development of New Antifungal Drugs

If antifungal ingredients are combined with immunomodulatory components of TCM, more ideal antifungal therapeutic effects may be obtained. However, there are still some problems in the development of TCM. Many TCM candidates have shown good antifungal activity in vitro, but few have shown consistent efficacy in animal models or clinical trials. This problem makes the application of TCM in antifungal therapy a great challenge.

There may be some reasons for this. First, the content of active ingredients in TCM is relatively low. We can use the technology of synthetic biology to genetically modify medicinal plants or use engineering yeast to increase the yield of effective components. Synthetic biology was applied to improve the production of natural products in many cases. For example, by introducing the *Artemisia annua* Linn. genes that encode the enzymes of the artemisinin biosynthetic pathway into yeast cells, we can improve the artemisinic acid yields from 1% to 25 g/L [129,130], although artemisinin production costs may increase as a result. The application of modern technology to improve the yield and economic benefits of the effective components can effectively solve some difficulties in the application of TCM.

Another possible reason is the relatively low efficacy and poor ADME properties of the active ingredient. We can modify the structure of the active small molecules of TCM by semi-synthesis or total synthesis, explore the structure–activity relationship, and select the molecules with better medicinal properties from the derivatives. TCM is a rich reservoir of biologically active natural products. It can provide us with many implications for the study of novel chemical structures with antifungal or immunomodulatory activities. We can screen for new lead compounds in this different TCM chemical pool. A variety of series of derivatives can be developed based on lead compounds to improve their efficacy, toxicity, and pharmacokinetic properties. By evaluating these derivatives, we can find antifungal candidates with high efficacy, low toxicity, and good ADME properties.

Currently, the development of TCM antifungal drugs is still in the stage of drug screening, and the mechanism of action of these bioactive molecules or mixtures is not clear. Future research should focus on the development of natural drugs with clear ingredients, a clear mechanism of action, and an optimized structure of active ingredients. It is hoped that new antifungal drugs with high efficiency and low toxicity can be developed.

## Figures and Tables

**Table 1:** Natural products from TCMs and their antifungal activities.

Natural Product	Source	Target Fungi	MIC (μg/mL) ^a^	FICI ^a^	References
dioscin 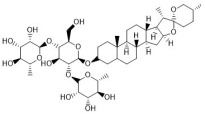	*Dioscoreaceae* family	*C. albicans* (2) ^b^	4	-	[14,15]
		*C. glabrata*	2	-	
		*C. parapsilosis*	4	-	
α-santalol 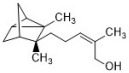	*Santalum* family	*T. rubrum*	12.5 (μg/disc ^c^)	-	[16,17]
eucarobustol E 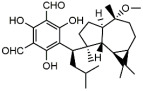	*Eucalyptus robusta*	fluconazole-susceptible *C. albicans* (10)	4–16	-	[18]
		fluconazole-resistant *C. albicans* (10)	32–128	-	[18]
asiatic acid 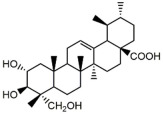	*Centella asiatica*	fluconazole-susceptible *C. albicans* (4)	64	0.75–1.00	[19]
		fluconazole-resistant *C. albicans* (4)	64–128	0.25-	[19]
carvacrol 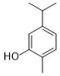	*Oreganum* family	*C. albicans*	250	0.374	[20,21]
thymol 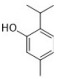	*Oreganum* family	*C. albicans*	500	1.062	[20,22]
		*C. neoformans* (10)	20–51	-	[23]
eugenol 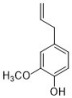	*Oreganum* family	*C. albicans*	1000	0.312	[20,24]
laurel essential oil	*Laurus nobilis*	*C. albicans* (2)	>4 (% *v*/*v*) ^d^	-	[25]
anise essential oil	*Pimpinella anisum*	*C. albicans* (6)	4 or >4 (% *v*/*v*)	-	[25]
oregano essential oil	*Thymus capitatus*	*C. albicans* (31)	0.0039–1, or <0.0019 (% *v*/*v*)	-	[25]
basil essential oil	*Ocimum basilicum*	*C. albicans* (11)	4 or >4 (% *v*/*v*)	-	[25]
lavender essential oil	*Lavandula latifolia*	*C. albicans* (15)	0.25–4 (% *v*/*v*)	-	[25]
mint essential oil	*Mentha spicata*	*C. albicans* (25)	2–4 (% *v*/*v*)	-	[25]
rosemary essential oil	*Rosmarinus officinalis*	*C. albicans* (2)	4 (% *v*/*v*)	-	[25]
rosemary extract	*Rosmarinus officinalis* L.	*C. albicans*	50,000	-	[26]
		*C. dubliniensis*	50,000	-	[26]
		*C. glabrata*	50,000	-	[26]
		*C. krusei*	50,000	-	[26]
		*C. tropicalis*	50,000	-	[26]
tea tree oil	*Melaleuca alternifolia*	*C. albicans* (44)	0.06–4 (% *v*/*v*)	0.25–1.25	[25,27]
terpinen-4-ol 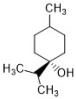	*Melaleuca alternifolia*	*C. albicans* (33)	0.06–0.25	0.250-0.252	[27,28]
grapefruit essential oil	*Citrus paradisi*	*C. albicans* (12)	0.0039–1 (% *v*/*v*)	-	[25]
turmeric essential oil	*Curcuma longa* L.	*M.gypseum* (2)	0.25 (% *v*/*v*), 6.25	-	[29,30]
		*T. mentagrophytes* (2)	0.25 (% *v*/*v*), 6.25	-	[29,30]
		*T. rubrum*	1.56	-	[30]
		*E. floccosum*	1.56	-	[30]
*Ar*-turmerone 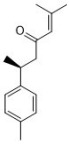	*Curcuma longa* L.	*M.gypseum*	7.81	-	[30,31]
		*T. mentagrophytes*	7.81	-	[30]
		*T. rubrum*	3.90	-	[30]
		*E. floccosum*	3.90	-	[30]
curcumin 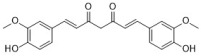	*Curcuma longa* L.	*fluconazole-susceptible Candida* species (27)	250–650	-	[32,33]
		*fluconazole-resistant Candida* species (11)	250–500	-	[32]
		*C. albicans*	64	-	[34]
		*C. tropicalis*	256	-	[34]
		*C. krusei*	256	-	[34]
		*C. parapsilosis*	>256	-	[34]
		*C. glabrata*	>256	-	[34]
		*C. dubliniensis* (2)	32	-	[34]
		*C. neoformans*	32	-	[34]
		*S. schenckii*	32	-	[34]
		*P. brasiliensis* (7)	0.5–32	-	[34]
		*A. fumigatus* (2)	>256	-	[34]
		*A. nomius*	>256	-	[34]
		*A. flavus*	>256	-	[34]
		*A. tamarii*	>256	-	[34]
		*A. terreus*	>256	-	[34]
		*A. clavatus*	>256	-	[34]
a blend of essential oils (3.53% of cinnamaldehyde, 3.53% of eugenol, 3.53% of carvacol, 1.04% of carotol, and 88.35% of *Camelina sativa* oil)	*Cinnamomum zeylanicum*, *Syzygium aromaticum*, *Origanum vulgare*, *Daucus carota*, and *Camelina sativa*	*C. albicans* (4)	0.02 (% *v*/*v*)	-	[35]
		*C. glabrata*	0.05 (% *v*/*v*)	-	[35]
		*C. tropicalis*	0.01 (% *v*/*v*)	-	[35]
silibinin 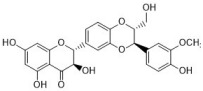	*Silybum marianum*	*C. albicans* (2)	19.3, 1024	-	[36,37,38]
		*C. krusei*	1024	-	[36]
		*C. tropicalis*	1024	-	[36]
		*A. flavus*	9.6	-	[37]
		*C. parapsilosis*	9.6	-	[37]
		*Malassezia Furfur*	19.3	-	[37]
		*Trichosporon* species	19.3	-	[37]
pinobanksin 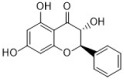	Chinese propolis	*C. albicans*	100	-	[39]
tectochrysin 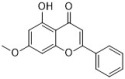	Chinese propolis	*C. albicans*	25	-	[39]
chrysin 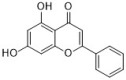	Chinese propolis	*C. albicans*	100	-	[39]
3-O-acetylpinobanksin 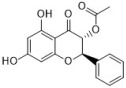	Chinese propolis	*C. albicans*	50	-	[39]
licochalcone A 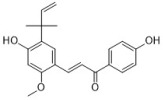	*Glycyrrhiza* family	*C. albicans* (4)	16.92–50.76	-	[40]
baicalein 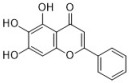	*Scutellaria baicalensis*	*C. albicans*	25	0.039	[41,42]
berberine 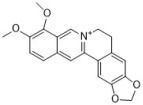	many herbs such as *Coptis chinensis* and *Mahonia aquifolium*	*C. albicans* (4)	80–160	0.017–0.127	[43,44]
		*C. krusei* (3)	10–20	-	[43]
		*C. glabrata* (3)	20–160	-	[43]
		*C. dubliniensis*	40	-	[43]
sanguinarine 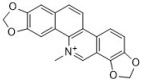	*Papaveraceae* family	*C. albicans* (11)	4, 37.5–50	-	[45,46]
		*C. neoformans*	64	-	[45]
chelerythrine 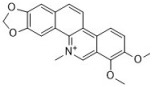	*Papaveraceae* family	*C. albicans* (3)	2–4	-	[45,47]
		*C. glabrata* (2)	16		[47]
		*C. krusei* (2)	16		[47]
		*C. tropicalis* (2)	8		[47]
		*C. neoformans*	64	-	[45]
plumbagin 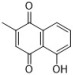	*Plumbago scandens*	*C. albicans*	0.78	-	[48]
		*C. neoformans*	8	-	[49]
bis-naphthoquinone 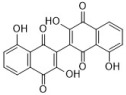	*Ceratostigma plumbaginoides*	*C. albicans*	0.09	-	[50]
shikonin 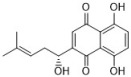	*Lithospermum erythrorhizon*	*C. albicans* (12)	2-8, >64	-	[51,52]
		*C. krusei*	4	-	[51]
		*C. glabrata*	8	-	[51]
		*C. tropicalis*	8	-	[51]
		*C. parapsilosis*	16	-	[51]
		*Saccharomyces cerevisiae*	4	-	[51]
		*C. neoformans*	8	-	[51]
		*T. cutaneum*	8	-	[51]
		*A. fumigatus*	>64	-	[51]
osthole 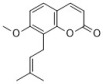	*Cnidii fructus*	*C. albicans* (52)	8–64, >64	0.04–0.37	[6,53]
ethyl caffeate 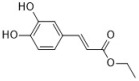	*Elephantopus scaber* L.	*C. albicans* (26)	64–256	0.047–0.375	[54]

^a^ MIC, minimum inhibitory concentration. FICI, fractional inhibitory concentration index; the numbers indicate the FICI of natural products combined with fluconazole against fungi. -, not mentioned in the report. ^b^ Numbers in the brackets indicate the number of strains tested. ^c^ In this case, MIC is defined as the concentration of the 0.5 mm inhibitory zone produced by the tested compound in a disc diffusion method. ^d^ In these cases, the unit of MICs is (% *v*/*v*) instead of μg/mL, since the agents are in liquid form.

## Data Availability

Data sharing not applicable.
